# Enhancing the Co-utilization of Biomass-Derived Mixed Sugars by Yeasts

**DOI:** 10.3389/fmicb.2018.03264

**Published:** 2019-01-22

**Authors:** Meirong Gao, Deon Ploessl, Zengyi Shao

**Affiliations:** ^1^Department of Chemical and Biological Engineering, Iowa State University, Ames, IA, United States; ^2^NSF Engineering Research Center for Biorenewable Chemicals (CBiRC), Iowa State University, Ames, IA, United States; ^3^The Ames Laboratory, Iowa State University, Ames, IA, United States; ^4^The Interdisciplinary Microbiology Program, Biorenewables Research Laboratory, Iowa State University, Ames, IA, United States

**Keywords:** mixed-sugar utilization, carbon catabolite repression, non-glucose transporters, consolidated bioprocessing, microbial consortium, cellulase, hemicellulase

## Abstract

Plant biomass is a promising carbon source for producing value-added chemicals, including transportation biofuels, polymer precursors, and various additives. Most engineered microbial hosts and a select group of wild-type species can metabolize mixed sugars including oligosaccharides, hexoses, and pentoses that are hydrolyzed from plant biomass. However, most of these microorganisms consume glucose preferentially to non-glucose sugars through mechanisms generally defined as carbon catabolite repression. The current lack of simultaneous mixed-sugar utilization limits achievable titers, yields, and productivities. Therefore, the development of microbial platforms capable of fermenting mixed sugars simultaneously from biomass hydrolysates is essential for economical industry-scale production, particularly for compounds with marginal profits. This review aims to summarize recent discoveries and breakthroughs in the engineering of yeast cell factories for improved mixed-sugar co-utilization based on various metabolic engineering approaches. Emphasis is placed on enhanced non-glucose utilization, discovery of novel sugar transporters free from glucose repression, native xylose-utilizing microbes, consolidated bioprocessing (CBP), improved cellulase secretion, and creation of microbial consortia for improving mixed-sugar utilization. Perspectives on the future development of biorenewables industry are provided in the end.

## Introduction

Legitimate concerns regarding the negative environmental impact and unsustainability of the petrochemical industry have resulted in extensive exploration in microbial production of fuels and chemicals (Chu and Majumdar, 2012). Currently, the majority of industrialized biochemical processes utilize crop sugar as substrate, which is non-ideal for a multitude of reasons, including minimal reductions in greenhouse gas emissions, food vs. fuel controversy, and uncompetitive margins compared to petrochemical counterparts (Carriquiry et al., 2011; Du et al., 2012; Latimer et al., 2014). Research foci have shifted toward utilizing lignocellulosic feedstock for second-generation fuel and chemical production because of its abundance, sustainability, and low price.

Although the prospect of second-generation fuel and chemical production offers multiple environmental and socioeconomical advantages, its current economic state renders the biorenewables industry incapable of competing with the petrochemical industry (Carriquiry et al., 2011). Multiple technical hurdles must be overcome to efficiently convert lignocellulosic biomass to biofuels/biochemicals.

Plant biomass is mainly comprised of cellulose, hemicellulose, and lignin with proportions of each component depending on the sources of plant biomass (Fengel and Wegener, 1983; Betts et al., 1991). After pretreatment and saccharification processing, the resulting biomass hydrolysates are mixtures of various hexoses and pentoses. Next to glucose, xylose and arabinose are the second and third most abundant sugars in most terrestrial plant biomass hydrolysates, respectively (Chandel et al., 2011; Kim et al., 2012). Efficient utilization of all types of sugars from plant biomass hydrolysates is essential for economic conversion of plant biomass to fuels and chemicals (Saha, 2003). Most microorganisms, including *Saccharomyces cerevisiae* and *Escherichia coli*, can efficiently utilize glucose as the optimal fermentation substrate, whereas non-glucose sugars such as xylose and arabinose are utilized at much lower efficiencies (Jojima et al., 2010).

Because of carbon catabolite repression, nearly all microorganisms utilize glucose preferentially over other sugars, resulting in extended fermentation periods and lower productivities (Zaldivar et al., 2001). Post-glucose depletion, other nutritional elements gradually become limited and certain fermentation end-products and/or side products that inhibit cell growth begin to accumulate in the culture media, eventually leading to the slow and incomplete fermentation of non-glucose sugars (Palmqvist et al., 1999; Graves et al., 2006). To address this issue, it is imperative to engineer microorganisms capable of simultaneous co-sugar utilization. Currently, microbial mixed-sugar conversion has been mostly studied in model microbial platforms such as *E. coli* and *S. cerevisiae* because of their well-understood physiology and genetic backgrounds, fast cell growth rates, and readily available genetic manipulation tools. Moreover, current commercial production of ethanol from sugarcane or cornstarch employs *S. cerevisiae*, and it was predicted that the integration of existing ethanol plants with engineered *S. cerevisiae* that produce biofuels from non-edible plant biomass can reduce the total cost by as much as 20% (Wooley et al., 1999; Peters et al., 2003).

This review describes recent advances in microbial conversion of mixed sugars from plant biomass, mainly focusing on *S. cerevisiae*. Various strategies used to ameliorate simultaneous mixed-sugar fermentation, including pathway introduction and optimization, discovery and engineering of novel specific transporters for special sugars, consolidated bioprocessing, and creation of microbial consortia, are discussed in detail. The vast majority of the studies discussed in this review analyzed bioethanol production. In these contexts, bioethanol is simply used as a reporter molecule for assessing mixed-sugar assimilation competence. In alignment with the broader scope laid out by the Department of Energy's Biomass Program (Aden et al., 2004), it is the hope that engineered strains can serve as microbial platforms to produce a wide array of biochemicals.

## History of Creating Xylose-Utilizing Strains

**Figure 1:**
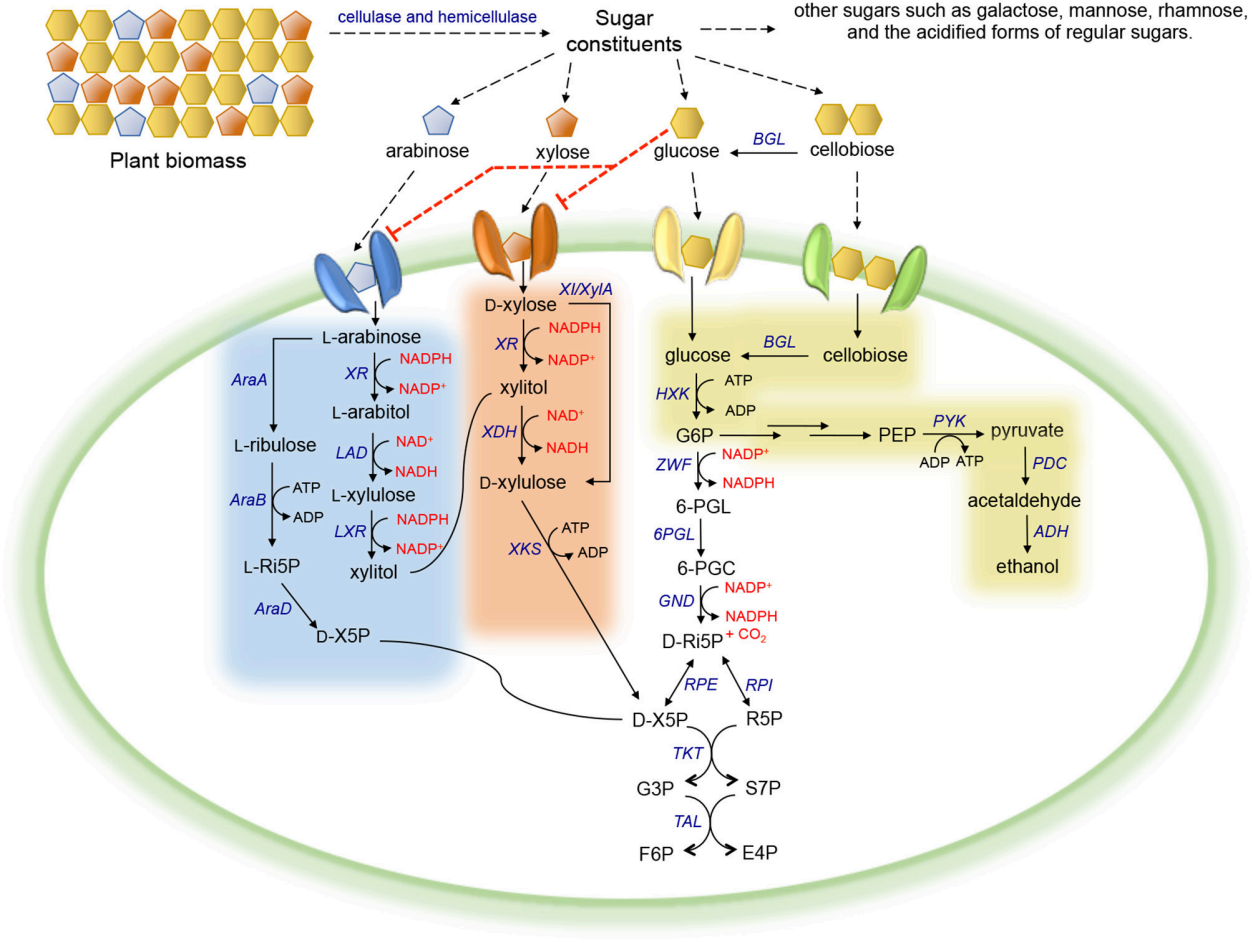
Carbohydrate metabolism in microorganisms. Red dotted line corresponds to inhibition. Abbreviation of metabolites—PEP, phosphoenolpyruvate; G6P, glucose-6-phosphate; 6-PGL, 6-phosphogluconolactone; 6-PGC, 6-phosphogluconate; D-Ri5P, D-ribulose-5-phosphate; D-X5P, D-xylulose-5-phosphate; R5P, ribose-5-phosphate; G3P, glyceraldehyde-3-phosphate; S7P, sedoheptulose-7-phosphate; F6P, fructose-6-phosphate; E4P, erythrose-4-phosphate; L-Ri5P, L-ribulose-5-phosphate. Abbreviation of enzymes—BGL, β-glucosidase; HXK, hexokinase; PYK, pyruvate kinase; PDC, pyruvate decarboxylase; ADH, alcohol dehydrogenase; ZWF, glucose-6-phosphate dehydrogenase; 6PGL, 6-phosphogluconolactonase; GND, 6-phosphogluconate dehydrogenase; RPI, ribose-5-phosphate isomerase; RPE, ribulose-5-phosphate epimerase; TKT, transketolase; TAL, transaldolase; XR, xylose reductase; XDH, xylose dehydrogenase; XKS, xylulokinase; XI/XylA, xylose isomerase; LAD, L-arabitol 4-dehydrogenase; LXR, L-xylulose reductase; AraA, L-arabinose isomerase; AraB, L-ribulokinase; AraD, L-ribulose-5-phosphate 4-epimerase.

Xylose, the second most abundant sugar in plant biomass, is metabolized by microorganisms mainly *via* two distinct routes (Figure 1). In native xylose-utilizing bacteria, some fungi, and plants, xylose is converted to D-xylulose by xylose isomerase (XI or XylA) in a single step (Schellenberg et al., 1984; Wilhelm and Hollenberg, 1984; Banerjee et al., 1994; Kristo et al., 1996; Rawat et al., 1996; Maehara et al., 2013), whereas in most innate xylose-utilizing fungi, a more complex alternative route consisting of two redox reactions exists. Xylose is first reduced to xylitol by a NADPH-preferred xylose reductase (XR). The resulting xylitol is then oxidized to D-xylulose by NAD^+^-dependent xylose dehydrogenase (XDH) (Chakravorty et al., 1962; Bruinenberg et al., 1984). Subsequently, D-xylulose derived from either pathway is phosphorylated by a xylulokinase (XKS) into D-xylulose 5-phosphate (D-X5P), which is then channeled into the pentose phosphate pathway (PPP) (Xue and Ho, 1990; Rodriguez-Pena et al., 1998; Hahn-Hägerdal et al., 2007).

Despite its broad industrial applications, *S. cerevisiae* cannot natively utilize xylose hydrolyzed from plant biomass, although gene homologs encoding XR, XDH, and XKS required for xylose metabolism are present in its genome (Hahn-Hägerdal et al., 2007). Overexpression of these native genes allowed for minimal cell growth on xylose (Toivari et al., 2004). Even after extensive evolution, *S. cerevisiae* strains with endogenous xylose metabolic pathways still could not metabolize xylose as efficiently as glucose (Attfield and Bell, 2006). This was mainly attributed to the imbalanced xylose-utilizing pathway, where the activities of XR and XDH were much lower compared to that of XKS.

To overcome this limitation, heterologous xylose-utilizing pathways were introduced into *S. cerevisiae*. First, despite its inability to ferment xylose, *S. cerevisiae* can grow on D-xylulose (Chiang et al., 1981), indicating that simply introducing a heterologous XI enables xylose utilization. The first highly functional *Piromyces* XI gene (Harhangi et al., 2003) that was introduced into *S. cerevisiae* conferred a specific growth rate of 0.005 h^−1^ on xylose under aerobic conditions (Kuyper et al., 2003, 2004). Continuous evolution in xylose media resulted in a mutant strain with improved growth rates of 0.18 h^−1^ under aerobic conditions and 0.03 h^−1^ under anaerobic conditions. The anaerobic ethanol yield from xylose was as high as 0.42 g g^−1^. Brat et al. identified the highly active *Clostridium phytofermentans* XI, a distant homolog of *Piromyces* XIs (Brat et al., 2009). Introducing a codon-optimized version into an industrial *S. cerevisiae* strain enabled an aerobic cell growth rate of 0.057 h^−1^ and anaerobic ethanol yield of 0.43 g g^−1^ when cultured in xylose. Subsequently, XIs from a series of species showing high similarities with *Piromyces* XI or *C. phytofermentans* XI were actively expressed in *S. cerevisiae* (Hahn-Hägerdal et al., 2007; Madhavan et al., 2009; Aeling et al., 2012; Hector et al., 2013; Peng et al., 2015). Particularly, through evolutionary engineering, XIs from *Prevotella ruminicola* (Hector et al., 2013), *Bacteroides vulgatus*, and *Alistipes* sp. HGB5 (Peng et al., 2015) displayed comparable enzyme activities to the best-reported XI from *Piromyces*. To date, many functionally expressed XIs in *S. cerevisiae* originated from mammal gut microflora, suggesting the evolutionary advantage of xylan-degrading bacteria present in mammalian guts. These findings have led to mammalian gut commensals serving as a repertoire for isolating novel genes involved in plant biomass degradation at standard temperatures (Peng et al., 2015).

In parallel to the XI pathway, the XR/XDH pathway has also been extensively studied in *S. cerevisiae* in the past few decades. Multitudinous efforts have been made to introduce genes encoding XR and XDH from natural xylose-assimilating species including *Scheffersomyces stipitis, Neurospora crassa*, and *Candida tenuis* into *S. cerevisiae*, leading to recombinant strains that grew aerobically on xylose media (Kotter et al., 1990; Kotter and Ciriacy, 1993; Tantirungkij et al., 1993; Jin et al., 2000; Petschacher and Nidetzky, 2008). However, xylose utilization was very slow in these recombinant strains and a large amount of xylitol accumulated, resulting in a low ethanol yield (Kotter and Ciriacy, 1993; Tantirungkij et al., 1993; Jin et al., 2000). Further investigation attributed this low xylose-utilizing efficiency to two main factors. The cofactor imbalance of XR and XDH was severe under oxygen-limited and anaerobic conditions, which resulted in excess formation of NADP^+^ and a shortage of NAD^+^. Because it lacked a transhydrogenase, *S. cerevisiae* cannot directly convert NADP^+^ to NAD^+^ (Hahn-Hägerdal et al., 2007). This imbalance was thought to result in xylitol by-product formation, pulling metabolic flux away from ethanol production (Karhumaa et al., 2007). Moreover, accumulation of PPP intermediates during xylose fermentation indicated that the capacity of the downstream PPP was constrained by PPP enzyme insufficiency (Kotter and Ciriacy, 1993). In the next two sections, strategies that have been implemented to improve xylose conversion in *S. cerevisiae* are discussed in detail.

## Strategies for Improving Xylose-Utilizing Pathway in *S. cerevisiae*

**Figure 2:**
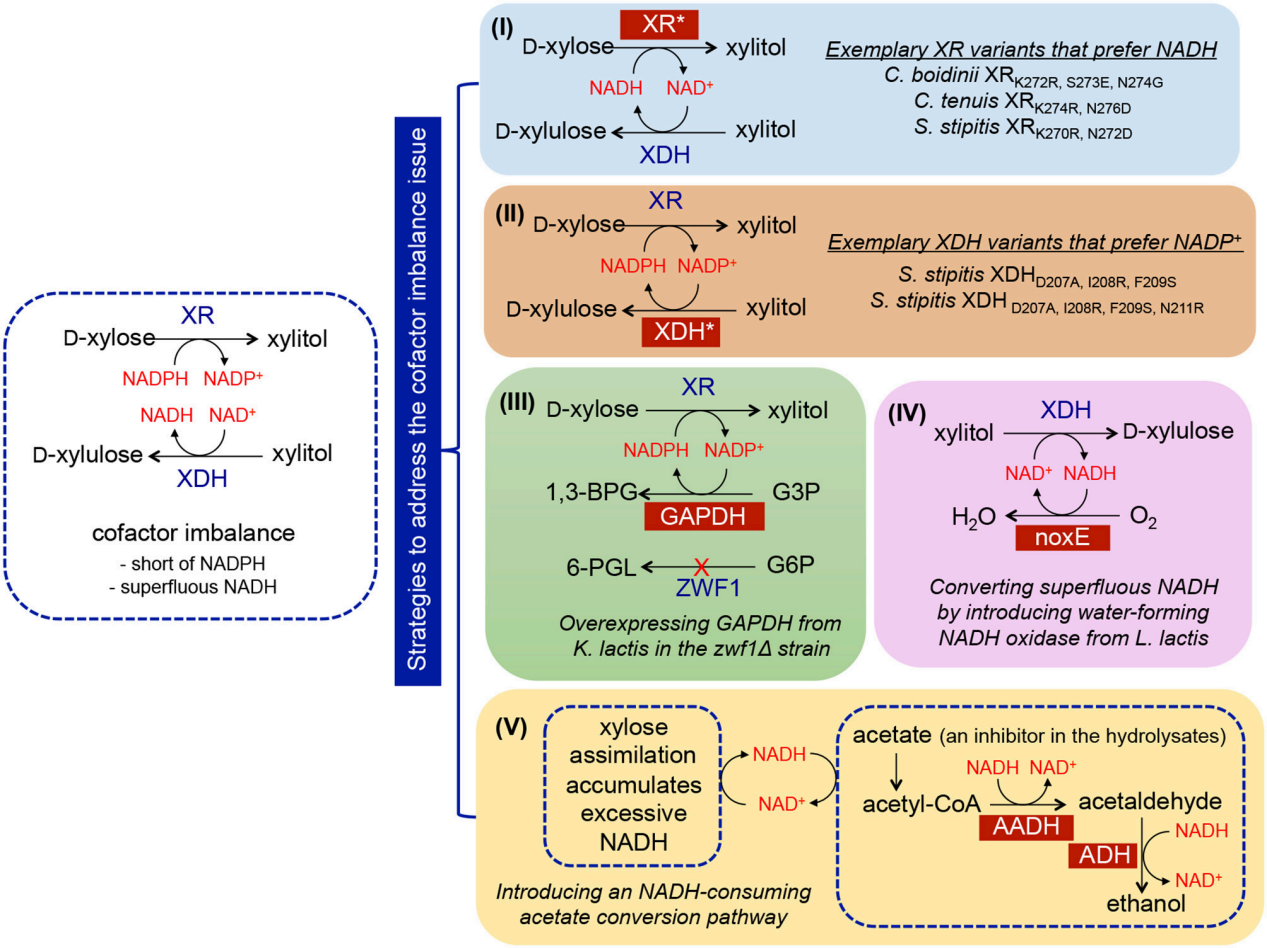
Implementation of metabolic engineering strategies in *S. cerevisiae* to enhance xylose utilization *via* ameliorating cofactor imbalance, including (I) discovery of NADH-preferred XR variants; (II) discovery of NADP^+^-preferred XDH variants; (III) enhancing NADPH regeneration through overexpressing NADP^+^-dependent GAPDH; (IV) enhancing NAD^+^ regeneration through overexpressing *noxE*; (V) improving NAD^+^ regeneration through the integration of acetate-consuming pathway. Abbreviation of metabolites−1,3-BPG, 1,3-bisphosphoglycerate; 6-PGL, 6-phosphogluconolactone; G6P, glucose-6-phosphate; G3P, glyceraldehyde-3-phosphate. Abbreviation of enzymes—*XR*, xylose reductase; *XDH*, xylose dehydrogenase; *ZWF*, glucose-6-phosphate dehydrogenase; *GAPDH*, glyceraldehyde-3-phosphate dehydrogenase; *noxE*, NADH oxidase; *AADH*, acetylating acetaldehyde dehydrogenase; *ADH*, alcohol dehydrogenase.

To mitigate the cofactor imbalance issue, numerous efforts have focused on either isolating novel NADH-preferred XR or switching the cofactor specificities of existing XRs (Kang et al., 2003; Khoury et al., 2009) (Figure 2, strategy I). For example, the cofactor specificity of *C. boidinii* XR was altered through computational design of the cofactor binding pocket, and subsequent characterization validated that seven mutants were more specific to NADH (Khoury et al., 2009). Four of the seven mutants completely lost activity when NADPH was the only available cofactor, and more than 10^4^-fold changes in substrate specificity from NADPH to NADH were observed for the mutant CbXR-K272R, S273E, N274G. Expression of the mutant XRs in *S. cerevisiae* containing the wild-type XDH resulted in increased xylose consumption rates, ethanol titers, and yields along with decreased xylitol levels (Jeppsson et al., 2006; Watanabe et al., 2007; Petschacher and Nidetzky, 2008; Bengtsson et al., 2009). Analogously, this strategy was applied to modify the specificity of XDH to NADP^+^ (Metzger and Hollenberg, 1995; Watanabe et al., 2005). Metzger et al. attempted to identify the NAD^+^-binding domain of *S. stipitis* XDH, and introduced a putative NADP^+^-recognition sequence (GSRPVC) of the alcohol dehydrogenase from *Thermoanaerobium brockii* (Metzger and Hollenberg, 1995). As a result, the mutant enzyme used both NAD^+^ and NADP^+^ as cofactors with equal apparent K_m_ values. The most successful work in changing the preference of XDH from NAD^+^ to NADP^+^ was performed by introducing triple (D207A/I208R/F209S) or quadruple (D207A/I208R/F209S/N211R) mutations into *S. stipitis* XDH (Watanabe et al., 2005) (Figure 2, strategy II). The resulting XDH variants displayed a shifted specificity toward NADP^+^, which was more than 4,500-fold higher than that displayed by the wild-type enzyme *in vitro*. Functional expression of the mutants in *S. cerevisiae* resulted in a maximal 86% decrease in xylitol accumulation coupled with a 41% increase in ethanol production compared to the control strain containing wild-type XDH (Watanabe et al., 2007).

A viable alternative strategy for enabling cofactor balance in *S. cerevisiae* containing the XR/XDH pathway is to enhance the regeneration of NADPH or NAD^+^. In wild-type yeast, NADPH is mainly regenerated *via* the oxidative portion of the PPP, in which two of the three enzymes involved in converting glucose-6-phosphate (G6P) to D-ribulose-5-phosphate (D-Ri5P) utilize NADP^+^ as a cofactor (Figure 1). However, this conversion is coupled with CO_2_ formation, inevitably resulting in significant carbon loss and low product yield (Verho et al., 2003). To reduce the heavy reliance on the PPP to regenerate NADPH, a yeast GPD1 gene, encoding NADP^+^-dependent D-glyceraldehyde-3-phosphate dehydrogenase (GAPDH) originated from *Kluyveromyces lactis*, was introduced into *S. cerevisiae* containing the XR/XDH pathway (Verho et al., 2003) (Figure 2, strategy III). Compared to *S. cerevisiae* containing endogenous NAD^+^-dependent GAPDH, overexpression of *K. lactis* GAPDH converted D-glyceraldehyde-3-phosphate (G3P) to 1, 3-bisphosphoglycerate using NADP^+^ as a cofactor, which is beneficial for both regenerating NADPH and reducing the accumulation of excessive NADH produced by endogenous GAPDH. When the *ZWF1* gene encoding glucose-6-phosphate dehydrogenase was simultaneously deleted, the *K. lactis* GAPDH became the major engine replenishing NADPH in recombinant *S. cerevisiae*, resulting in higher ethanol production rates and yields from xylose compared to those in the control strain. The same strategy was applied to improve NAD^+^ availability by heterologously expressing *Lactococcus lactis* NADH oxidase in *S. cerevisiae* containing the XR/XDH pathway, which mediated the conversion of NADH and oxygen to NAD^+^ and water (Heux et al., 2006; Vemuri et al., 2007; Zhang et al., 2012; Hou et al., 2014) (Figure 2, strategy IV). Introduction of the water-forming NADH oxidase mitigated this cofactor imbalance by converting excess intracellular NADH to NAD^+^ (Heux et al., 2006; Vemuri et al., 2007). During xylose fermentation, the recombinant strain overexpressing NADH oxidase from *L. lactis* demonstrated a 69.63% decrease in xylitol accumulation, a 53.85% reduction in glycerol production, and a 39.33% increase in ethanol yield (Zhang et al., 2012).

In addition to NADH oxidase, other NAD^+^-regenerating pathways were explored. For example, Wei et al. confirmed that in yeast, acetate can be converted to ethanol catalyzed by the endogenous acetyl-CoA synthetase and alcohol dehydrogenase (ADH) in conjunction with the heterologous acetylating acetaldehyde dehydrogenase (AADH) using NADH as a cofactor (Wei et al., 2013) (Figure 2, strategy V). A group of AADH homologs was tested, including bifunctional proteins isolated from *E. coli, Piromyce*s Sp. E2, and *Clostridium beijerinckii* (catalyzing the conversion of acetyl-CoA to acetaldehyde followed by subsequent conversion to ethanol), and the proteins involved in ethanolamine or 4-hydroxy-2-ketovalerate catabolism from *E. coli*. Introduction of these homologs into *S. cerevisiae* harboring the XR/XDH pathway enabled simultaneous acetate and xylose consumption under anaerobic conditions. The highest improvement was achieved by overexpressing the bifunctional *E. coli* AdhE, which led to 17% and 21% increases in ethanol yield and productivity, respectively. This improvement was attributed to the increased regeneration of NAD^+^ (by 50%) and decreased levels of glycerol and xylitol byproducts. The superiority of this system is conferred by co-utilization of xylose and acetate, the latter of which is a ubiquitous inhibitor present in plant biomass hydrolysates and serves as a redox sink for consuming surplus NADH.

Although D-xylulose, the product of XR/XDH-catalyzed conversion, can be metabolized by *S. cerevisiae*, it is consumed much more slowly than glucose (Senac and Hahn-Hagerdal, 1990; Yu et al., 1995). Numerous independent studies indicated that merely introducing XI or XR/XDH resulted in substantial D-xylulose accumulation in *S. cerevisiae* (Jin and Jeffries, 2003; Kuyper et al., 2004; Madhavan et al., 2009). These observations suggested insufficient activity of endogenous XKS. To overcome this inadequacy, genes encoding XKS from multiple sources were expressed in recombinant *S. cerevisiae* harboring either the XI or XR/XDH pathway (Moniruzzaman et al., 1997; Johansson et al., 2001; Toivari et al., 2001; Madhavan et al., 2009; Zhou et al., 2012). Notably, excessive XKS activity may increase metabolic burden and cause excessive ATP consumption, impairing cell growth and diminishing ethanol yield (Johansson et al., 2001; Jin et al., 2003). In this regard, fine-tuned XKS activity is essential for optimizing xylose fermentation in recombinant *S. cerevisiae* strains. As a result, moderate XKS activity coupled with the high activity of XR and XDH was optimal for xylose conversion (Eliasson et al., 2001; Matsushika and Sawayama, 2008).

## Strategies for Improving PPP in *S. cerevisiae* for Efficient Xylose Assimilation

The PPP is the entry point for integrating D-xylulose into central carbon metabolism. Therefore, the activity of PPP enzymes is crucial for efficient pentose assimilation (Hahn-Hägerdal et al., 2007). Because of the low capacity of the non-oxidative portion of the PPP (Fiaux et al., 2003), *S. cerevisiae* containing a xylose-assimilating pathway accumulated PPP intermediates, including 6-phosphogluconate (6-PGC), D-X5P, D-Ri5P, ribose-5-phosphate (R5P), and sedoheptulose-7-phosphate (S7P) (Senac and Hahn-Hagerdal, 1990; Kotter and Ciriacy, 1993; Matsushika et al., 2013) (Figure 1). In addition to strategies of elevating the expression level of the endogenous transketolase (TKT1) and transaldolase (TAL1), researchers have attempted to overexpress the homologs of the key enzymes involved in the PPP to improve pathway flux in xylose-fermenting yeast. Some previous studies reported that overexpression of *S. stipitis* TKT1 in *S. cerevisiae* containing the XR/XDH pathway did not affect xylose utilization, and in some cases, imposed significant metabolic burden and drastically extended the cell-doubling time on xylose (Metzger and Hollenberg, 1994; Walfridsson et al., 1995). In contrast, other studies indicated that overexpression of either the endogenous or heterologous TAL1 from *S. stipitis* in xylose-utilizing *S. cerevisiae* enhanced cell growth on xylose (Walfridsson et al., 1995; Jin et al., 2005). Specifically, *S. cerevisiae* transformed with XR, XDH, XKS, and TAL1 isolated from *S. stipitis* grew twice as fast on xylose and produced 70% more ethanol compared to the control strain overexpressing only XR/XDH/XKS (Jin et al., 2005). Moreover, two parallel studies reported the overexpression of genes encoding the entire non-oxidative PPP, including ribose-5-phosphate isomerase (RPI), ribulose-5-phosphate epimerase (RPE), TKT1, and TAL1, in the recombinant xylose-fermenting *S. cerevisiae*, along with deletion of the gene encoding aldose reductase (GRE3) that mediates unwanted production of xylitol (Karhumaa et al., 2005; Kuyper et al., 2005). The resulting strains demonstrated improved cell growth rate (0.22 h^−1^ and 0.17 h^−1^) and enhanced ethanol production (0.43 g g^−1^ and 0.29 g g^−1^) on xylose (Figure 1).

## Strategies for Enabling Growth on Arabinose

Arabinose is the second most abundant pentose in hemicellulose (Madhavan et al., 2012). Microorganisms metabolize arabinose to D-X5P, an intermediate of the PPP, via two distinct pathways (Figure 1) (Richard et al., 2003). Bacteria utilize arabinose isomerase (AraA), L-ribulokinase (AraB), and L-ribulose-5-phosphate 4-epimerase (AraD) to convert arabinose to D-X5P through intermediates L-ribulose and L-Ri5P (Wisselink et al., 2007). The disparate fungal alternative arabinose pathway converts arabinose to D-X5P through 5 steps *via* four intermediates (i.e., L-arabitol, L-xylulose, xylitol, and D-xylulose), and the corresponding reactions are catalyzed by aldose reductase (GRE3 or XR), L-arabitol 4-dehydrogenase (LAD), L-xylulose reductase (LXR), XDH, and XKS (Chiang and Knight, 1961; Witteveen et al., 1989; Richard et al., 2001, 2002). In natural arabinose-assimilating microorganisms (e.g., *S. stipitis* and most *Candida* species), L-arabitol accumulation resulted in very low ethanol titers and yields (Mcmillan and Boynton, 1994; Dien et al., 1996). Moreover, the unclear genetic background and unavailable genetic manipulation tools limit the direct application of most natural arabinose-utilizing microorganisms as production hosts in the biorenewables industry. To date, studies of arabinose utilization were mainly conducted in model microbial hosts.

Similar to xylose, arabinose does not support the growth of wild-type *S. cerevisiae* (Hahn-Hägerdal et al., 2007). Attempts have been made to enable arabinose utilization in *S. cerevisiae* by introducing the fungal arabinose pathway (Richard et al., 2003; Verho et al., 2004; Bettiga et al., 2009; Bera et al., 2010). Despite improved arabinose utilization through pathway optimization, the accumulation of L-arabitol indicated an imbalance in redox cofactors during arabinose fermentation in the engineered *S. cerevisiae*. The fungal arabinose pathway comprises two NADPH-dependent reductions and two NAD^+^-dependent oxidations. Although the expression of an NADH-dependent XR from *Ambrosiozyma monospora* mitigated the co-factor imbalance, excessive L-arabitol accumulation resulted in low ethanol yield (Verho et al., 2004; Bettiga et al., 2009).

Unlike its fungal counterpart, the bacterial arabinose pathway evades the cofactor imbalance and represents a viable alternative to enable arabinose utilization in *S. cerevisiae*. However, direct introduction of the bacterial arabinose pathway from *E. coli, Bacillus subtilis* or *Lactobacillus plantarum* into *S. cerevisiae* did not support robust cell growth on arabinose until extensive evolutionary engineering was applied (Sedlak and Ho, 2001; Becker and Boles, 2003; Wisselink et al., 2007, 2009; Sanchez et al., 2010; Wang et al., 2013, 2017). For example, Wisselink et al. built a recombinant *S. cerevisiae* strain (IMS001) containing the arabinose pathway from *L. plantarum, Piromyces sp*. strain E2 XI, and yeast *XKS* gene, coupled with overexpression of endogenous non-oxidative PPP genes, including *TAL1, TKL1, RPE1*, and *RPI1* (Wisselink et al., 2007). However, IMS001 did not grow on arabinose, although expression of the arabinose pathway was verified by real-time PCR, albeit some genes expressed at very low levels. Only after evolutionary engineering, could the resulting strain IMS002 anaerobically grow on arabinose with a sugar consumption rate of 0.7 g h^−1^ g^−1^_drybiomass_ and ethanol productivity of 0.29 g h^−1^ g^−1^_dryweight_. In anaerobic glucose/arabinose co-fermentation, arabinose was utilized upon glucose depletion at a rate of 0.42 g h^−1^ g^−1^_drybiomass_ with an ethanol yield of 0.42 g g^−1^. Subsequently, IMS002 was subjected to another round of evolutionary engineering to regain the xylose-assimilating ability lost during long-term selection for improved arabinose fermentation (Sanchez et al., 2010). The resulting strain converted a mixture of glucose, xylose, and arabinose to ethanol, achieving a yield of 0.43 g g-1, with a minimal byproduct accumulation (xylitol and L-arabitol) Very recently, a novel arabinose pathway from *Pediococcus pentosaceus*, a native arabinose-utilizing bacterium, and an arabinose transporter from *Spathaspora passalidarum* were isolated and introduced into *S. cerevisiae* (Caballero and Ramos, 2017). After one round of evolutionary engineering, arabinose was metabolized aerobically. A second round of evolutionary adaption under anaerobic conditions was carried out and the resulting strain demonstrated anaerobic growth and an ethanol production yield as high as 0.43 g g^−1^. To date, the arabinose-utilizing efficiency and ethanol yield have been largely improved, albeit glucose repression on arabinose utilization remains unchanged (Wisselink et al., 2007; Wang et al., 2017).

## Exploration of Novel Pentose Transporters

Initial efforts devoted to improving xylose assimilation were mainly focused on increasing intracellular pentose utilization. Although these efforts enhanced intracellular pentose flux into central metabolism, it was recognized that sugar uptake constrained achievable xylose assimilation (Jojima et al., 2010; Young et al., 2010, 2014). Because *S. cerevisiae* lacks xylose-specific transporters, the native hexose transporters, including HXT1, HXT2, HXT4, HXT5, HXT7, and GAL2, are responsible for mediating xylose transport (Hamacher et al., 2002; Sedlak and Ho, 2004; Saloheimo et al., 2007; Young et al., 2011). However, these hexose transporters possess 10-100 fold higher affinity for glucose compared to other sugars. This resultant glucose preference extends mixed-sugar fermentation process time (Van Vleet and Jeffries, 2009; Young et al., 2010; Kim et al., 2012). Therefore, mining and/or engineering xylose-specific transporters insensitive to glucose is essential for economic conversion of mixed sugars present in biomass hydrolysates.

The first non-native xylose transporters successfully expressed in *S. cerevisiae* were GXF1 and GXS1 from *Candida intermedia* (Leandro et al., 2006). Both transporters supported cell growth on xylose in the hexose transporter-null *S. cerevisiae* strain EBY.VW4000, which was transformed with a xylose-utilizing pathway. EBY.VW4000, deprived of 18 hexose transporters and three maltose transporters that transport hexoses, has been used as a platform for characterizing pentose transporters (Wieczorke et al., 1999). Although kinetic studies indicated that GXF1 is primarily a hexose transporter, its affinity toward xylose is 3-fold higher than those of native *S. cerevisiae* hexose transporters (Leandro et al., 2006; Runquist et al., 2009). Taking advantage of this property, GXF1 was expressed in xylose-fermenting *S. cerevisiae* strains, leading to faster xylose uptake rates (1.4–4-fold) and improved cell growth rates (1.7–4-fold) in xylose fermentation compared to the strain harboring only a xylose-utilizing pathway. Other putative xylose transporters from diverse organisms, including plants, algae, fungi, and bacteria, were expressed in *S. cerevisiae* to improve xylose uptake (Saloheimo et al., 2007; Hector et al., 2008; Katahira et al., 2008; Runquist et al., 2009, 2010; Du et al., 2010; Young et al., 2011). Because of potential improper localization or misfolding, many of these proteins did not enable xylose uptake in *S. cerevisiae* despite their high homology to known xylose transporters. For example, using GXS1 as a query, a study aimed to discover novel xylose transporters from native xylose-assimilating yeasts, *S. stipitis* and *N. crassa* (Du et al., 2010). Among a total of 18 putative xylose transporters, An25 and Xyp29 were identified as xylose-specific. Their expression on the membranes of *S. cerevisiae* was confirmed and xylose was accumulated intracellularly because of the scarcity of an efficient xylose-utilizing pathway in the engineered *S. cerevisiae*. In a subsequent study, a collection of 36 putative sugar transporters was thoroughly characterized for their substrate acceptance profiles in the EBY.VW4000 strain expressing XR and XDH from *S. stipitis* (Young et al., 2011). Growth-based assays demonstrated that three transporters, XUT1 and XUT3 from *S. stipitis* and XylHP from *Debaryomyces hansenii*, transported xylose efficiently and demonstrated higher xylose preference than other transporters in the hexose transporter-null strain background.

Despite the performance of these transporters in xylose media, all of these transporters were subjected to glucose repression in mixed-sugar fermentation or suffered from low xylose-uptake efficiency (Hector et al., 2008; Katahira et al., 2008; Runquist et al., 2009, 2010; Du et al., 2010; Young et al., 2011). In this regard, to overcome CCR and promote efficient biochemical production, numerous independent studies were conducted to reshape the existing transporters *via* rational design or evolutionary engineering (Young et al., 2012, 2014; Farwick et al., 2014; Nijland et al., 2014; Shin et al., 2015; Li et al., 2016; Reider Apel et al., 2016; Wang et al., 2016). Presumably, glucose repression can be exerted outside of the xylose-binding pocket. The very first transporter engineering work was performed by Young et al. (2012). Starting with *C. intermedia* GXS1 and *S. stipitis* XUT3, five mutants with improved xylose uptake activities were generated by employing a directed evolution method. Kinetic studies indicated that some mutated transporters obtained increased affinity to xylose (lower K_m_) and higher uptake rates (higher V_max_), with the best mutant amplifying the yeast cell growth rate on xylose by 1.7-fold compared to its corresponding wild-type. Relieved CCR and enhanced sugar assimilation were observed in mixed-sugar fermentation. The phenylalanine residue at position 40 of GXS1 and glutamine residue at position 538 of XUT3 were found to largely contribute to influencing the transportation characteristics.

Led by the observation that sugar transporter preference does not require a trade-off for its efficiency, multiple primary hexose transporters were engineered to be xylose-exclusive (Farwick et al., 2014; Nijland et al., 2014; Young et al., 2014; Shin et al., 2015; Li et al., 2016; Reider Apel et al., 2016; Wang et al., 2016). After evaluating 46 heterologous transporters, a conserved protein motif G-G/F-XXX-G surrounding the previously reported GXS1-F40 residue was recognized to dominate the transporters displaying high efficiency in xylose transport (Young et al., 2014). Through a series of saturation mutagenesis followed by rational mutagenesis, four xylose-exclusive transporters variants, CiGXS1F^38^I^39^M^40^, SsRGT2F^38^, SsRGT2M^40^, and ScHXT7I^39^M^40^M^340^, were obtained. The four mutants conferred robust cell growth on xylose in EBY.VW4000, albeit still inhibited by glucose, presumably *via* allosteric binding. Nearly concurrently, Farwick et al. built an easy yet precise xylose-specific transporter screening system by disrupting the first step of the glycolytic pathway (Farwick et al., 2014). Through growth-based screening under mixed-sugar conditions, a combinatorial strategy of evolutionary engineering and site-specific mutagenesis led to the discovery of several mutations that contributed to mitigating glucose inhibition. Specifically, simply altering threonine 219/213 and asparagine 376/370 of *S. cerevisiae* GAL2/HXT7 resulted in mutant transporters possessing extraordinary properties, including attenuated or completely abolished glucose affinity, enhanced xylose affinity, and decreased glucose competitive repression. This is the first study in which primary hexose transporters were engineered to be both glucose-insensitive and xylose-specific. Subsequently, various independent studies reported that hexose transporters such as *S. cerevisiae* HXT36 (Nijland et al., 2014), HXT11 (Shin et al., 2015), HXT7 (Reider Apel et al., 2016), *C. intermedia* GXS1 (Li et al., 2016), and pentose transporter [e.g., *N. crassa* An25 (Wang et al., 2016)] were engineered to relieve glucose inhibition and improve xylose uptake. These transporter-engineering studies suggested that direct evolution and combinatorial mutagenesis are potent protein engineering tools for expanding the collection of pentose-favoring or pentose-specific transporters.

To facilitate uptake and *in situ* intracellular conversion of xylose, a very intriguing study was recently conducted to construct an artificial enzyme-transporter complex in *S. cerevisiae* (Thomik et al., 2017). After a series of optimizations, a scaffold-based complex successfully recruited the heterologously expressed *C. phytofermentans* XI to the endogenous GAL2 localized on the membrane, which did not negatively affect the activities of both proteins. Because of the ameliorated substrate transportation and channeling, the xylose consumption rate and ethanol titer were remarkably increased with substantially diminished accumulation of xylitol, the undesired byproduct.

## Native xylose-utilizing yeasts

Most recent studies used EBY.VW4000 or its derivatives as the transporter-screening platform. However, this strain grows slowly because of its defects in meiosis and centromere segregation caused by large chromosome translocations (Solis-Escalante et al., 2015). In this regard, EBY.VW4000 is a good strain for transporter screening but not for industrial mixed-sugar conversion. Moreover, as described previously, xylose fermentation in engineered *S. cerevisiae* is accompanied by co-factor imbalance and subsequently low metabolic flux. Thus, a highly promising alternative to engineering industrial friendly model hosts to efficiently utilize mixed sugars is ameliorating innate xylose-utilizing microorganisms (Alper and Stephanopoulos, 2009). The CUG clade is a group of nonconventional yeasts that translate the CUG codon to serine rather than leucine (Santos et al., 2011). Unlike *S. cerevisiae*, most CUG clade yeasts can uptake and metabolize xylose much more efficiently. Specifically, *S. stipitis* and *S. passalidarum* are particularly intriguing because of their ability to convert xylose to ethanol at high yields and efficiencies (Van Vleet and Jeffries, 2009; Su et al., 2015). For instance, a newly isolated *S. passalidarum* strain, CMUWF1-2, converted xylose to ethanol with a yield of 0.43 g ethanol g^−1^ xylose and exhibited minimal glucose repression in a mixed-sugar assay (Rodrussamee et al., 2018). However, because of the dominant non-homologous end joining mechanism for repairing DNA double strand breaks and a lack of genetic manipulation tools, the exploration pace of CUG clade yeast has been limited, preventing their applications in industrial biofuel/biochemical production. Recent advances in synthetic biology have greatly expedited progress in engineering non-conventional yeasts (Lobs et al., 2017). For example, a series of genetic manipulation tools for *S. stipitis* were reported, including strong and constitutive promoters and terminators (Gao et al., 2017), a stable episomal expression plasmid created by successful isolation of native centromeres (Cao et al., 2017a,c), and CRISPR-enabled precise genome editing tools (Cao et al., 2017b). Through utilization of these tools, studies have demonstrated the possibility of engineering *S. stipitis* to an efficient platform in the near future for producing a specific group of biochemicals [i.e., shikimate pathway derivatives (Suastegui and Shao, 2016; Gao et al., 2017)] or biofuels directly from biomass hydrolysates containing hexoses and pentoses.

In addition to the CUG clade yeasts, oleaginous yeasts, such as *Yarrowia lipolytica* and *Psedozyma hubeiensis*, also exhibit native mixed-sugar consumption capabilities. Employing comprehensive metabolic and transcriptomic analysis strategies, a recent study unraveled the genes responsible for xylose and cellobiose uptake and metabolism in *Y. lipolytica* (Ryu et al., 2016). Simultaneous mixed-sugar utilization was achieved, which was attributed to mild glucose repression in conjunction with strong carbon catabolite activation for growth on xylose and cellobiose. In another exhaustive screening of 1,189 yeast isolates, Tanimura et al. demonstrated *P. hubeiensis* IPM1-10 was able to co-utilize glucose, xylose, and arabinose in the artificial hydrolysate and accumulated high amounts of lipid (Tanimura et al., 2016). These findings suggest the importance of broadening the current collection of microbial species for efficient fermentation of multiple sugars in biomass hydrolysates.

## Transportation of Other Carbon Sources

Researchers have also attempted to develop an alternative conversion scheme, which is to co-ferment oligosaccharides and pentoses (Yang et al., 2014). In contrast to *S. cerevisiae*, which cannot metabolize cellodextrins natively, the cellulolytic fungus *N. crassa* grows on cellodextrins because of its high-affinity cellodextrin transport system (Galazka et al., 2010). Thus, to bypass extracellular glucose suppression on hexose/pentose co-utilization, the integration of the newly discovered cellodextrin transport system (Tian et al., 2009; Galazka et al., 2010) in conjunction with a xylose-utilizing pathway facilitated simultaneous fermentation of cellobiose and xylose in engineered *S. cerevisiae* (Li et al., 2010; Ha et al., 2011). Specifically, genes encoding the cellodextrin transporter and an intracellular β-glucosidase were co-expressed in *S. cerevisiae* containing a functional xylose pathway. The resulting strain produced 48 g L^−1^ ethanol from a mixture of 10 g L^−1^ glucose, 80 g L^−1^ cellobiose, and 40 g L^−1^ xylose in 60 h fermentation, more productive than *S. stipitis* cultured under the same conditions. The major advantage of the integrated system resides in the fact that the intracellular glucose hydrolyzed from cellobiose did not repress extracellular xylose transportation, therefore enabling co-sugar uptake. Moreover, the synergistic effects of cellobiose and xylose co-utilization greatly improved ethanol yield and productivity (Ha et al., 2011). Similarly, co-fermentation of cellodextrin and galactose was successfully accomplished in *S. cerevisiae*, and an appreciable increase in ethanol productivity was observed during mixed-sugar fermentation (Ha et al., 2011). Taken together, this unique strategy advances the economic production of fuels and chemicals from plant biomass.

In addition to its viability on cellobiose, *N. crassa* also grows well on xylodextrin (e.g., xylobiose, xylotriose, and xylotetraose), owing to its innate xylodextrin transport and utilization pathway (Li et al., 2015). Reconstituting this pathway in *S. cerevisiae* enabled slow cell growth on xylodextrin. An ortholog of *N. crassa* xylodextrin transporter, ST16 from *Trichoderma virens*, enabled *S. cerevisiae* to grow more rapidly on xylodextrins as sole carbon source (Zhang et al., 2017). Moreover, ST16 was not subjected to cellobiose inhibition, making it a promising candidate for the co-fermentation of cellobiose and xylodextrins.

Similar to xylose, arabinose is exogenous to *S. cerevisiae*, although it can be assimilated by wild-type *S. cerevisiae*, facilitated mainly by the hexose transporter GAL2 (Becker and Boles, 2003; Subtil and Boles, 2011). In contrast to xylose transporters, heterologous arabinose transporters have not been extensively studied and previous studies mainly focused on introducing a functional arabinose utilization pathway into *S. cerevisiae*. Several studies showed that GAL2 overexpression in *S. cerevisiae* strains enhanced arabinose fermentation under various culture conditions (Becker and Boles, 2003; Wang et al., 2013). However, GAL2 is a non-specific, low-affinity arabinose transporter subjected to glucose repression (Kou et al., 1970; Subtil and Boles, 2011). Therefore, to improve arabinose uptake in *S. cerevisiae*, putative arabinose transporters from multiple species were characterized over the past several years. Verho et al. identified and characterized the first eukaryotic genes encoding arabinose-exclusive transporters, LAT1 and LAT2 from *A. monospora*, a native arabinose-assimilating yeast (Verho et al., 2011). Expression of LAT1 and LAT2 in the *S. cerevisiae* hexose transporter-null strain containing the fungal arabinose-utilizing pathway conferred cell growth on arabinose. Shortly thereafter, additional arabinose transporters were isolated, including AraT from *S. stipitis, S. passalidarum*, and *Penicillium chrysogenum*, Stp2 from *Arabidopsis thaliana*, LAT-1 from *N. crassa* and *Myceliophthora thermophila*, and AXT1 from *Kluyveromyces marxianus* and *Pichia guilliermondii* (Subtil and Boles, 2011; Benz et al., 2014; Knoshaug et al., 2015; Li et al., 2015; Caballero and Ramos, 2017; Bracher et al., 2018). Although these reported arabinose transporters improved arabinose uptake in *S. cerevisiae*, their activities in glucose/arabinose-containing cultures were either not tested or were found to be inhibited by glucose. Therefore, future targets regarding mining arabinose transporters should focus on relieving hexose repression while improving intracellular metabolic flux.

## Consolidated Bioprocessing

One major challenge in the efficient conversion of plant biomass to fuels/chemicals involves deconstructing complex components to release the entrained fermentable sugars. This is inherently difficult because plants have evolved recalcitrance to withstand numerous forces attempting to jeopardize their structure (Olson et al., 2012). To overcome this recalcitrance and ferment plant biomass efficiently, dedicated biomass pretreatment processes have been developed, including chemical hydrolysis, pyrolysis, and enzymatic catalysis. These additional processing steps are partially responsible for the high production cost of second-generation fuels/chemicals (Olson et al., 2012). Consolidated bioprocessing (CBP) aims to simplify and reduce the cost of biorefining by combining cellulase production, lignocellulose degradation, and sugar fermentation to desired products in one process without pretreatment or enzyme supplementation. CBP requires a microbial host that can both break down and utilize lignocellulose, as well as produce desired products at high yields/titers. Unfortunately, no natural hosts can efficiently perform both tasks. Therefore, to engineer a host for CBP, two major strategies have been developed, including: (i) engineering a fuel/chemical-producing workhorse such as *E. coli* or *S. cerevisiae* to utilize plant feedstocks and (ii) engineering a plant biomass-consuming organism (e.g., *Aspergillus, Clostridium, Cellulomonas*) to produce desired fuels/chemicals. This section mainly describes recent advances in engineering *S. cerevisiae* to utilize minimally processed plant biomass as feedstocks.

**Figure 3:**
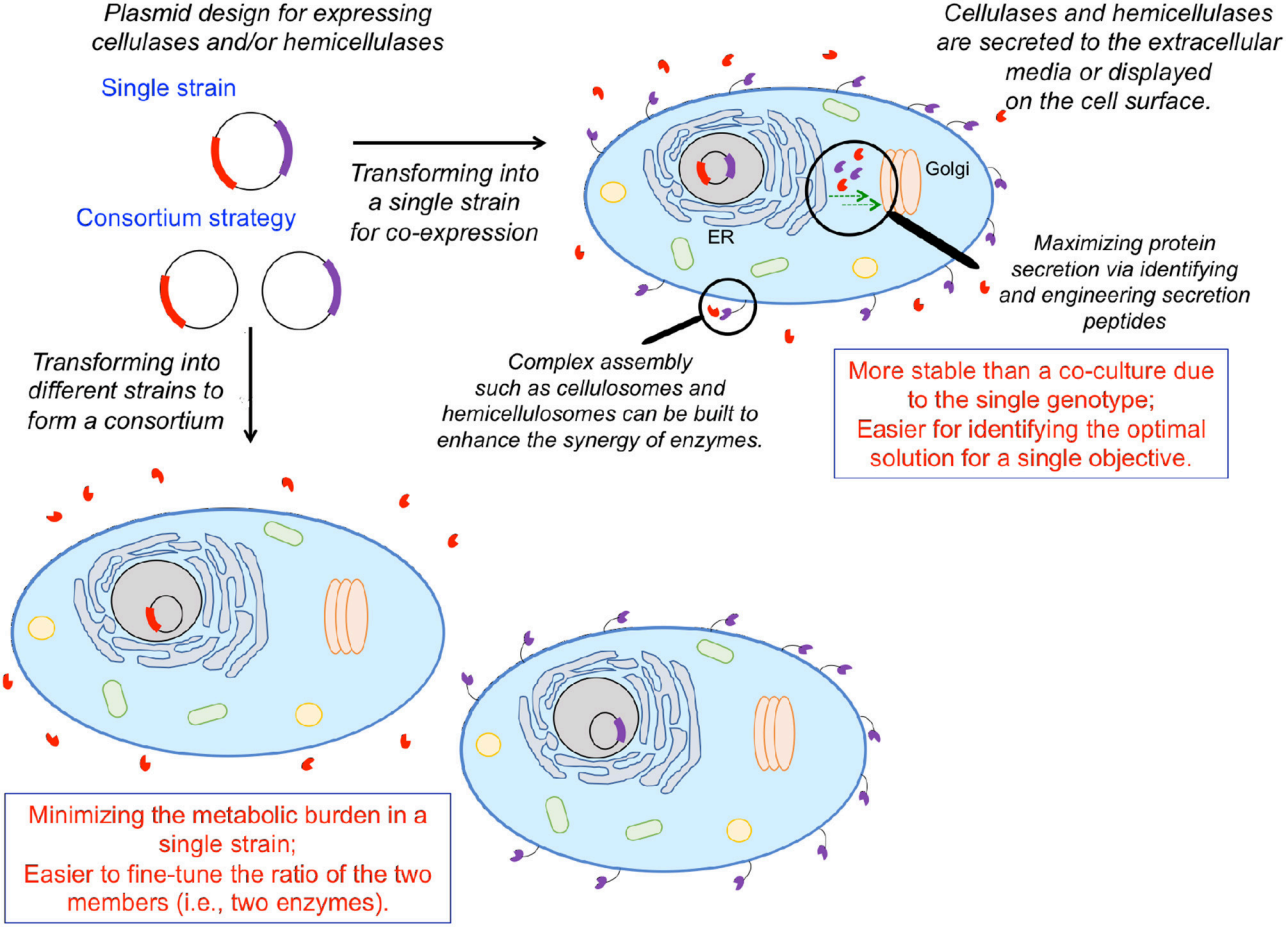
Solubilizing biomass to fermentable sugar substrates for biochemical production *via* heterologous expression of cellulases and hemicellulases. One can either engineer a single organism to express multiple cellulolytic/hemicellulolytic enzymes or employ a consortium strategy, engineering multiple organisms to each express a single enzyme. Enzymes can either be expressed on the cell surface, or secreted to the extracellular medium, depending on the application. Heterologous protein expression can be enhanced *via* identifying/engineering secretion tags and facilitating transport either from the endoplasmic reticulum to the Golgi, or the Golgi to the cell surface. Individual enzymes can be brought into spatial proximity *via* assembly of complex structures mimicking cellulosomes and hemicellulosomes to coordinate enzymatic activities.

Engineering *S. cerevisiae* to simultaneously break down plant biomass and ferment the released carbohydrates to desired products requires the integration of lignocellulolytic capabilities. Numerous organisms exhibit lignocellulolytic activity, including various bacteria, fungi, crustaceans, and insects (Gírio et al., 2010). Various cellulases/hemicellulases enable these organisms to depolymerize plant biomass into fermentable sugars. Numerous engineering efforts to integrate lignocellulolytic activity in *S. cerevisiae* and other hosts have focused on heterologous expression of such enzymes either on the cell surface or through secretion to the extracellular media (Figure 3). Cellobiose can be hydrolyzed to glucose by β-glycosidase (Figure 1). Rooyen et al. expressed two β-glucosidases, BGL1 from *Saccharomycopsis fibuligera* and BglA from *Aspergillus kawachii*, on the cell surface of *S. cerevisiase*, enabling it to grow anaerobically on cellobiose as the sole carbon source (van Rooyen et al., 2005). Unlike glucose, cellobiose does not repress the uptake of other sugars such as xylose and galactose (Li et al., 2010). Thus, *S. cerevisiae* has been engineered to co-utilize cellobiose and xylose or cellobiose and galactose in the past decade. Multiple studies employed a ubiquitous strategy: expression of a surface-displayed β-glycosidase to enable cleavage of cellobiose into glucose with the resulting glucose being rapidly ingested by yeast cells, functioning to maintain a low extracellular glucose concentration to ameliorate glucose repression on ingestion of other sugars (Katahira et al., 2006; Nakamura et al., 2008; Saitoh et al., 2010). As a proof of concept, the first xylose/cellobiose co-fermenting yeast strain was built *via* integrating an intracellular xylose pathway and a β-glycosidase tethered to the cell surface in a single strain (Katahira et al., 2006). The resulting strain assimilated xylose in the presence of a high cellobiose concentration as rapidly as that in pure xylose media (Nakamura et al., 2008). The simultaneous utilization of xylose and cellobiose resulted in 35.9 g L^−1^ of ethanol from 50 g L^−1^ cellobiose and 50 g L^−1^ xylose with a yield of 0.36 g g^−1^ and a productivity of 0.50 g L^−1^ h^−1^, which was 34% higher than that of glucose/xylose co-fermentation. Subsequently, an improved strategy was employed to construct a more efficient cellobiose/xylose-utilizing strain starting from an industrial yeast (OC2-HUT) (Saitoh et al., 2010). Specifically, two copies of the xylose pathway and four copies of genes encoding cell-surface-displayed β-glycosidase were integrated into the genome. The resulting strain consumed 90 g L^−1^ cellobiose and 60 g L^−1^ xylose in 48 h, increasing ethanol titer, yield, and productivity to 57.4 g L^−1^, 0.38 g g^−1^, 1.2 g L^−1^ h^−1^, respectively. Because excessive extracellular cellobiose hydrolysis and non-efficient glucose transportation into the cell would result in glucose accumulation in the medium and further repress xylose uptake (Kim et al., 2012), a balanced glucose uptake rate and surface displayed β-glycosidase activity must be carefully maintained.

Similarly, Jeon et al. expressed BGL1 along with EngD, an endoglucanase from *Clostridium cellulovorans* in *S. cerevisiae*, and promoted secretion of these enzymes into the extracellular media by fusing them to the α-mating factor secretion signal peptide. The resulting strain produced ethanol from β-glucan, achieving 80.3% of the theoretical yield (Jeon et al., 2009). An engineered xylose-utilizing *S. cerevisiae* strain co-displaying xylanase II from *Trichoderma reesei* and β-xylosidase from *Aspergillus oryzae* produced 7.1 g L^−1^ ethanol directly from birchwood xylan after a 62-h fermentation (Katahira et al., 2004). Sakamoto et al. co-expressed a cellulase (β-glucosidase from *Aspergillus aculeatus*) and hemicellulases (the xylanase II and β-xylosidase enzymes previously mentioned Katahira et al., 2004) in a xylose-utilizing *S. cerevisiae* strain. The resulting strain produced 8.2 g L^−1^ ethanol from rice straw hydrolysates after a 72-h fermentation (Sakamoto et al., 2012). Although these studies confirm that *S. cerevisiae* can be engineered to utilize cellulose/hemicellulose, the activities of the expressed enzymes must be coordinated to realize the full potential of CBP.

**Figure 4:**
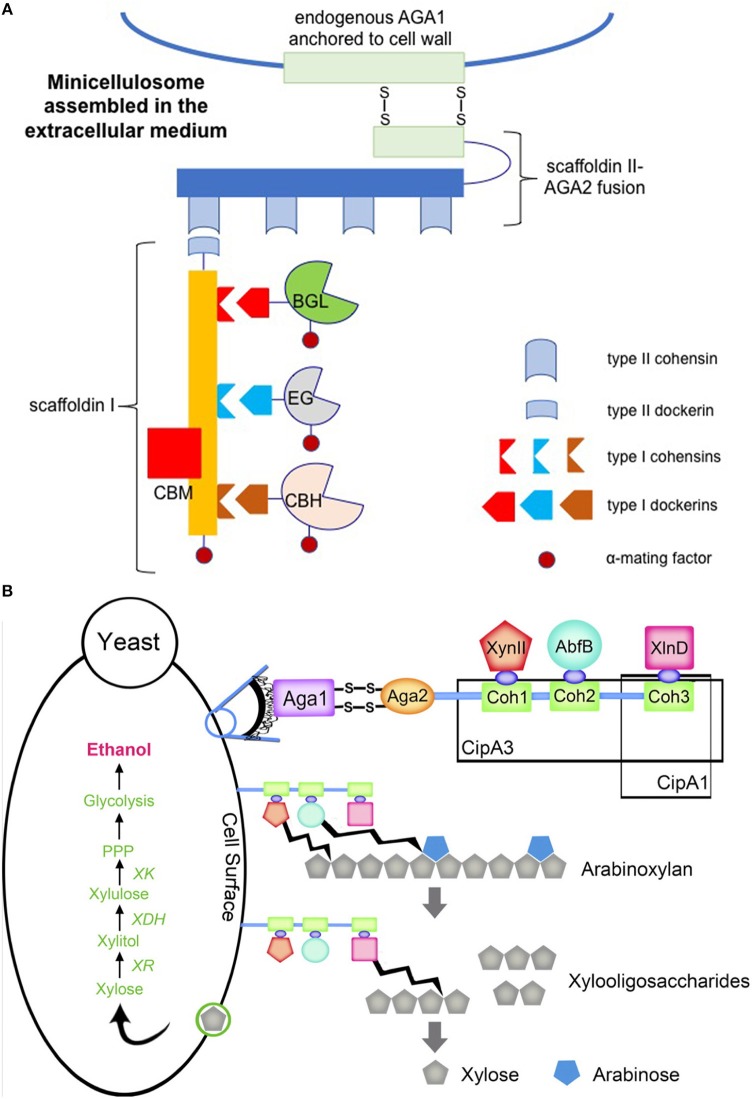
Engineered cellulosome **(A)** and hemicellulosome **(B)** in *S. cerevisiae*. **(A)** Three cellulases from *Clostridia* were heterologously expressed in *S. cerevisiae*, including endoglucanase (EG), cellobiohydrolase (CBH), and β-glucosidase (BGL). Each cellulase contained a type I dockerin specific to one of three type I cohesins present on scaffoldin I which was expressed by the recombinant strain. Fusion of these four components to α-mating factor enabled secretion to the extracellular medium to prevent intracellular assembly. Expression of a scaffoldin II-a-agglutinin adhesion subunit (AGA2) fusion tethered scaffoldin I-cellulase complexes to the cell wall *via* type II dockerin-cohesin interactions. Scaffoldin II-AGA2 anchored to the cell wall *via* formation of two disulfide bridges between AGA2 and endogenous a-agglutinin anchorage subunit (AGA1). The image was prepared according to the previous study carried out by Fan et al. (2012) **(B)** Minihemicellulosomes were expressed in *S. cerevisiae* carrying the xylose utilizing pathway. The miniscaffoldin CipA1 contained one type I cohesion (Coh3), whereas the miniscaffoldin CipA3 contained three type I cohesions (Coh1, Coh2, and Coh3). Each miniscaffoldin was tethered to the cell wall *via* AGA1 and AGA2 interaction, analogous to the cellulosome structure in **(A)**. The endoxylanase (XynII), arabinofuranosidase (AbfB), and β-xylosidase (XlnD) were tethered to the miniscaffolidns *via* type 1 dockerin-cohesin interactions. The original figure published in Sun et al. (2012) was shown here with a copyright permission.

In nature, some lignocellulolytic organisms organize their cellulases into complexes known as cellulosomes (Bayer et al., 2008). The function of the cellulosome is to mediate binding to lignocellulosic biomass and coordinate enzymatic activities for efficient biomass solubilization. The discovery and characterization of cellulosomes has inspired engineering efforts to mimic these structures with the aim of synergistically increasing heterologous enzyme activities. Fan et al. engineered a *S. cerevisiae* strain that displayed a mini-cellulosome on its cell surface (Fan et al., 2012) (Figure 4A). This was accomplished by first secreting three different cellulases from mesophilic *Clostridia* along with scaffoldin I, which contains the necessary type I cohesins for cellulase anchoring using α-mating factor. Scaffoldin II was then cell surface-displayed *via* fusion expression with the smaller subunit (69 amino acids) of the a-agglutinin receptor, which forms two disulfide bonds with the larger subunit (725 amino acids) that is endogenously produced and then covalently attached to the yeast cell wall matrix. Scaffoldin I was also fused with a type II dockerin at the C-terminus, which subsequently bound to one of the four repeating type II cohesions carried by scaffoldin II. This enabled tethering of the engineered mini-cellulosome to the cell surface. The resulting strain directly fermented Avicel and phosphoric acid swollen cellulose (PASC) to ethanol, achieving titers of 1.41 and 1.09 g L^−1^ after 4-day fermentation, respectively.

Similarly, Sun *et al*. constructed two separate mini-hemicellulosomes by expressing two different miniscaffoldins, one with a single cohesion module and the other with three cohesion modules, from *Clostridium thermocellum* in a xylose-utilizing *S. cerevisiae* strain (Sun et al., 2012) (Figure 4B). These miniscaffoldins were then attached to the cell surface *via* the a-agglutinin adhesion receptor. Heterologous expression of three hemicellulases with C-terminal dockerins mediated non-specific anchoring of the enzymes on the miniscaffoldins. With three possibilities at each cohesion, 27 different trifunctional minihemicellulosomes were engineered. In contrast, the miniscaffoldin equipped with only one cohesion module randomly adhered to one of the three hemicellulases with C-terminal dockerins. The trifunctional mini-hemicellulosome design exhibited 1.39-fold higher arabinoxylan conversion activity than the mini-hemicellulosomes bound to only one of the hemicellulases, demonstrating the synergistic benefit of placing the three hemicellulases in proximity. Fan et al. observed analogous results in their cellulosome system, noting that the trifunctional system exhibited higher activity than the sum of three unifunctional systems, resulting in a 3.6-fold increase in the ethanol titer (Fan et al., 2012).

## Strategies for Improving Cellulase Secretion

One major culprit inhibiting the secretion of heterologous enzymes and efficient surface display is the yeast secretory pathway. Misfolding and post-translational modifications differing from the native host can jeopardize catalytic activity (Boer et al., 2000), prevent transport out of the Golgi, and/or result in degradation in the endoplasmic reticulum (ER) (Lambertz et al., 2014). Overexpression of a heterologous protein can also pose an excessive burden to a cell's protein folding and secretion machinery. Recently, Tang *et al*. overexpressed genes involved in vesicle trafficking from the ER to the Golgi and from the Golgi to the plasma membrane, enhancing the secretion and surface display of BGL1 and endoglucanase from *C. thermocellum* in *S. cerevisiae* (Tang et al., 2017) (Figure 3). This work also suggested that the rate-limiting step in the yeast secretory pathway is protein-specific. It was found that overexpression of genes associated with transport from the ER to the Golgi had a more pronounced effect on endoglucanase secretion, whereas overexpression of genes associated with transport from the Golgi to the cell membrane had a more pronounced effect on BGL1 secretion. This work demonstrated that modifying the processes of vesicle budding, tethering, and fusion is a useful strategy for increasing enzyme secretion and surface display. Another strategy for increasing the secretion of heterologous protein in *S. cerevisiae* is to fuse optimal secretion signals to the desired protein. Bae et al. developed a high-throughput screening method for selecting a translational fusion partner (TFP) for optimal protein secretion (Bae et al., 2015). By manipulating the invertase (catalyzing the hydrolysis of sucrose into fructose and glucose) fusion partner trap system, secretion of a desired protein was coupled with the growth of *SUC2*-disrupted *S. cerevisiae* on sucrose-only media. Lee et al. implemented this screening method to select TFPs for four different cellulases and then expressed each of the four cellulases with the accompanying TFP in a *S. cerevisiae* strain (Lee et al., 2017). These four strains were then co-fermented, producing ethanol from pre-treated rice straw at approximately 3-fold higher productivity than the control wild-type strain in the culture supplemented with 10 filter paper units commercial cellulases per gram of glucan. To implement such consortia in practical industrial applications, determining co-fermentation seed ratios (which dictates enzyme ratios) is necessary for optimizing synergistic enzymatic activity. Variance in growth rates amongst engineered strains can make this task cumbersome, presenting a technical burden to practical applications (Lee et al., 2017). Further characterization of the optimal enzyme ratios and enzyme-secreting strains are required for efficient lignocellulose solubilization.

Recently, Liu et al. proposed a novel strategy for lignocellulose saccharification. Scanning electron microscopy imaging revealed that cellulase-displaying *S. cerevisiae* cells adhered to PASC, a phenomenon not observed in cellulase-secreting *S. cerevisiae*. Additionally, the cellulase-displaying strain achieved a 9% higher ethanol yield than the cellulase-secreting strain. Adhesion of the cellulase-displaying strain mediated a “tearing” effect on PASC, increasing the rate of breakdown compared to free enzyme hydrolysis obtained from the cellulase-secreting strain (Liu et al., 2016). Interestingly, adhesion was not observed and the ethanol yields were about the same for both strains when Avicel was used as the substrate. These results indicate that the rough, high surface area properties of the amorphous PASC enables adhesion, whereas the crystalline Avicel cannot promote such a mechanism. Promoting cell adhesion to biomass is a new engineering approach to enhance saccharification rates.

## Applying Consortia in Mixed-Sugar Conversion

Engineering microbial communities represents a new frontier of synthetic biology and has garnered increasing attention in recent years (Brenner et al., 2008). Microbial co-culture systems can perform complex tasks *via* labor division. Unlike monocultures, consortium enables the partitioning of multi-step metabolic pathways to each member of the consortium. Not only does this compartmentalization alleviate excessive metabolic burden on a single member, but it also provides the crucial ability to optimize segmented pathways to avoid compromise, thus minimizing byproduct accumulation (Zhang et al., 2015). In nature, communities respond more robustly to environmental fluctuations such as nutrient limitation, and this feature may be particularly useful for the fermentation of biomass hydrolysates when sugar choices and concentrations are shifting (Brenner et al., 2008). In this section, the applications of both natural and synthetic consortia for enhancing mixed-sugar consumption and improving biochemical production are discussed. Although this review mainly focuses on yeast engineering, a few interesting bacterium consortium examples are included below.

Microbial consortia are ubiquitous in nature, and synergistic interactions among community species are established after long-term evolution in specific environments (Brenner et al., 2008). Applying natural consortia in mixed-sugar utilization is beneficially associated with non-sterile fermentation at reduced operation costs (Zuroff and Curtis, 2012). For example, thermophilic bacterium consortia enriched from geothermal spring were evolved to produce hydrogen from glucose/xylose mixtures and hydrolysates of oil palm trunk with yields of 375 and 301 mL H_2_ g^−1^ sugar consumed, respectively (Hniman et al., 2011). The dominant species in the consortia were *Thermoanaerobacterium* sp., *Thermoanaerobacter* sp., *Caloramater* sp., and *Anoxybacillus* sp. In addition to the major product hydrogen, multiple byproducts were detected, including butyric acid, acetic acid, lactic acid, and butanol. Although natural consortia are durable under dynamic culture conditions because of their high self-tunability and have been applied to complex tasks such as wastewater treatment and lignocellulosic biomass deconstruction, their uncontrollability limits their wide application in producing specific biochemicals at high titers (Zuroff and Curtis, 2012). The physiology and genetic background of members of natural consortia are not well-characterized, further complicating determination of the interaction mechanisms in the microbial community.

To address this issue, significant progress has been made in the design and characterization of synthetic microbial consortia because of their relatively simple interplays (Zuroff and Curtis, 2012). The simplest design is to build a synthetic community with strains from the same species, which avoids growth incompatibility issues, including temperature, pH, nutrition requirement, and growth rate. Eiteman et al. developed an *E. coli*/*E. coli* community for simultaneous conversion of glucose and xylose to a mixture of valuable products, including formate, lactate, ethanol, and succinate (Eiteman et al., 2008). One of the two community members was engineered to be glucose-exclusive with its native XI deleted, while the other was xylose-selective with three genes involved in glucose uptake and phosphorylation disrupted. Rather than adversely competing with one another, the two *E. coli* strains acted in concert to co-utilize sugar mixtures, leading to a higher sugar consumption rate than the monocultures. The distinguishing hallmark of the consortium strategy is the robustness and flexibility provided by the system. Fed-batch fermentation indicated that this consortium adjusted the ratio of each strain based on fluctuations in the feed sugar concentration. For example, when xylose concentration is higher than glucose in the feed stream, the xylose-consuming strain would be dominant, and vice versa. Subsequently, the same group further extended the consortium approach to the simultaneous consumption of glucose, xylose, and arabinose along with acetate, which is a growth inhibitor typically present in lignocellulosic hydrolysates (Xia et al., 2012). Four *E. coli* strains engineered to be single substrate-selective were co-inoculated, and synchronous utilization of four substrates was achieved, albeit the xylose and arabinose consumption rates were decreased by 14 and 11%, respectively, compared to those in the wild-type strain.

Employing the analogous rationale to explore the synchronous conversion of glucose/xylose/arabinose mixtures, a consortium system consisting of three *S. cerevisiae* specialists was established recently (Verhoeven et al., 2018). Both xylose- and arabinose-exclusive specialists were constructed *via* disrupting hexose phosphorylation followed with adaptive evolution. Despite the rapid consumption of glucose and arabinose by the three-strain co-culture, xylose utilization was severely impaired, possibly due to (by-)product inhibition. Only after substantial anaerobic laboratory evolution was xylose utilization significantly improved, eventually leading to the simultaneous depletion of all three sugars. Moreover, the evolved three-strain consortium was more stable and robust than a pentose-fermenting yeast monoculture during long-term cultivation. Very recently, a kinetic model was constructed to provide guidelines for mixed-sugar fermentation *via S. cervisiae* co-cultures. This model, which incorporated pure culture models, product inhibition effects, initial substrate concentrations, and inoculum sizes, accurately predicted independent experimental results (Chen et al., 2018). Implementation of the model prediction enabled co-fermentation of 60 g L^−1^ cellobiose and 20 g L^−1^ xylose in a *S. cerevisiae/S. cerevisiae* co-culture.

**Figure 5:**
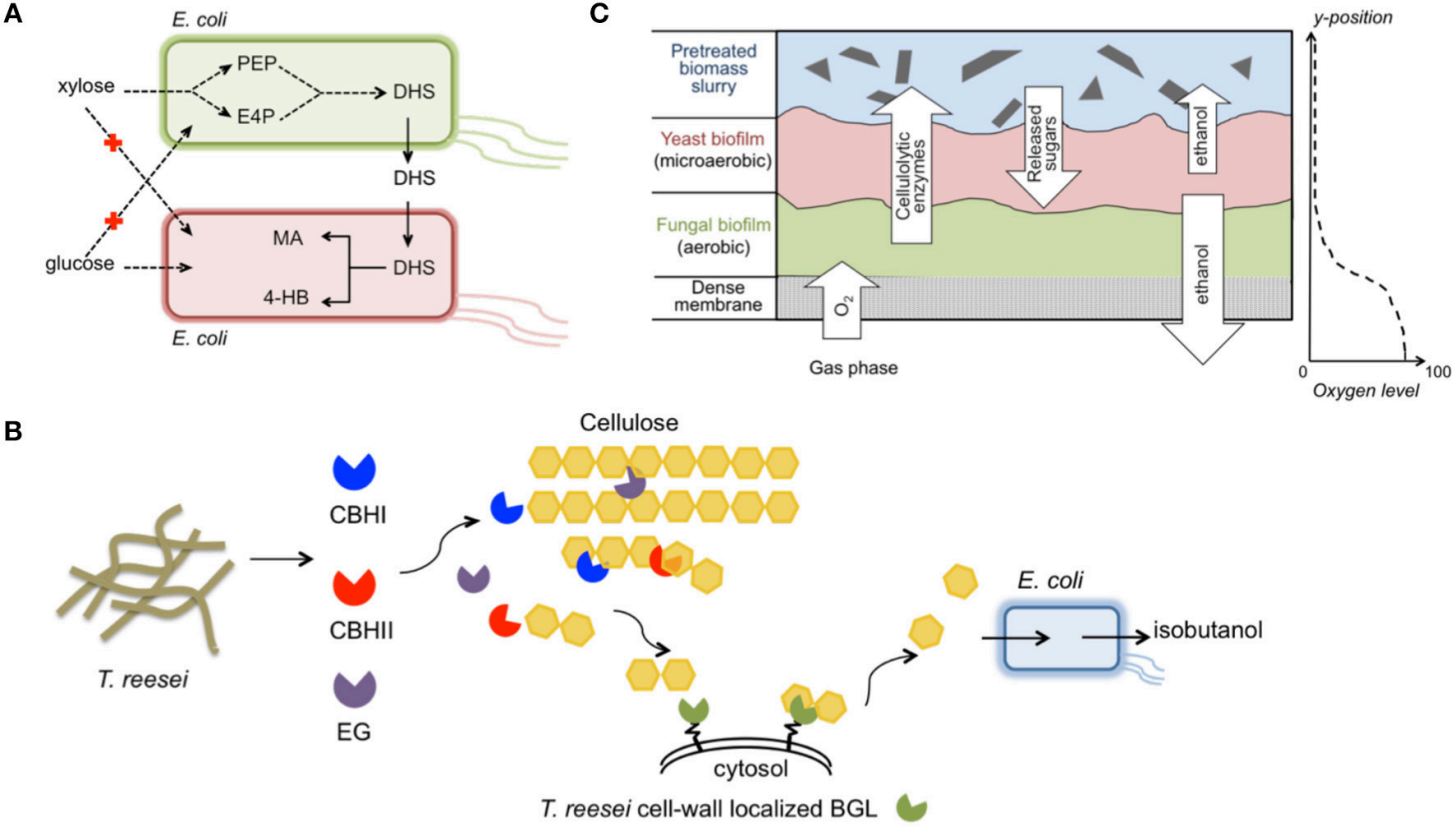
Exemplary engineered microbial consortia. **(A)** An *E. coli*/*E. coli* consortium for improved production of MA and 4HB from a glucose/xylose mixture (Zhang et al., 2015); **(B)** A *T. reesei*/*E. coli* consortium for direct conversion of biomass hydrolysates to isobutanol (Minty et al., 2013); **(C)** A *T. reesei*/*S. cerevisiae*/*S. stipitis* biofilm membrane reactor integrating aerobic cellulolytic enzyme production and anaerobic ethanol accumulation (Brethauer and Studer, 2014). The images were prepared according to the previous individual studies. Abbreviation of metabolites— PEP, phosphoenolpyruvate; E4P, erythrose-4-phosphate; DHS, dehydroshikimate; MA, *cis, cis*-muconic acid; 4-HB, 4-hydroxybenzoic acid; CBHI, cellobiohydrolase I; CBHII, cellobiohydrolase II; EG, endoglucanase; BGL, β-glucosidase.

A stable *E. coli*/*E. coli* integrated system that produced high-value compounds from glucose/xylose mixtures was constructed and optimized (Zhang et al., 2015) (Figure 5A). To overcome the lingering high leakage of pathway intermediates and non-efficient sugar utilization, the multi-step *cis, cis*-muconic acid (MA) synthesis pathway was split into two independent *E. coli* strains. One strain preferred xylose and contained the shikimate pathway, which catalyzed the conversion of xylose to the intermediate dehydroshikimate (DHS). The other strain preferred glucose and harbored the exogenous DHS-to-MA synthesis pathway together with a DHS transporter. As a result, glucose/xylose co-consumption was achieved, eliminating carbon source competition in the community. Additionally, the microbial community improved the MA titer to 4.7 g L^−1^ with a yield as high as 0.35 g g^−1^ sugar mixture. Because of the tunability and dynamic growth balance exhibited by the two constituent members, the community was more robust for handling varying mixed-sugar concentrations. Finally, the co-culture concept was extended to produce 4-hydroxybenzoic acid (4HB) using the DHS-producing strain and a new strain that converted DHS to 4HB. Through simple independent engineering of the new strain, a final titer of 2.3 g L^−1^ 4HB was obtained, and the yield of 0.11 g g^−1^ was equivalent to the highest reported yield achieved in previously reported glucose fermentations (Barker and Frost, 2001).

Compared to single-species consortia, manipulating communities comprising multi-species is more complicated. This is attributed to the community instability caused by different growth rates and difficulty in building cell-cell communication across species. Solving these instability issues would be advantageous, since multi-species consortia possess unique advantages. A wider array of mixed species offer higher flexibility and capability to achieve more complex tasks. Additionally, a better understanding of multi-species consortia is beneficial for future studies to break the confines of model microorganisms and exploit non-model hosts, particularly those with desirable phenotypes but are recalcitrant to genetic modifications. Numerous studies have focused on developing microbial consortia of *S. cerevisiae* and a native pentose-fermenting yeast for efficient hexose/pentose co-utilization. For example, under oxygen-limited and continuous culture conditions, the co-cultured *S. stipitis* and respiratory-deficient *S. cerevisiae* system converted 35.0 g L^−1^ glucose and 10.4 g L^−1^ xylose and displayed enhanced ethanol yield at 0.42 g g^−1^_sugars_ (Laplace et al., 1993). Such a co-cultured yeast system was further optimized *via* adjusting the agitation rate, temperature, and continuous dilution rate (Taniguchi et al., 1997; Delgenes et al., 1998; Suriyachai et al., 2013). To identify the key determinants of the performance of this *S. cerevisiae*/*S. stipitis* co-culture system, a dynamic metabolic flux balance model was constructed (Hanly and Henson, 2013) and an 11% improvement in ethanol productivity was observed by adjusting the aeration level and inoculation concentrations predicted by the model. Slow xylose uptake and conversion were determined as the key factors restraining ethanol production by this *S. cerevisiae*/*S. stipitis* co-culture.

A unique synthetic fungal-bacterial mixed population was developed for isobutanol production directly from plant biomass (Minty et al., 2013) (Figure 5B). Isobutanol is a highly sought-after next-generation biofuel with advantageous properties as a gasoline substitute, including higher energy density, lower hygroscopicity, and lower volatility compared to ethanol (Atsumi et al., 2008). This consortium system integrated cellulase secretion, sugar saccharification, and fermentation *via* combining *T. reesei*, an active cellulase-producing host, and *E. coli* that was engineered to convert sugars to isobutanol. The dedicated design maintained a very low glucose concentration in the medium, analogous to ideal CBP conditions. A comprehensive ordinary differential equation model established, which included 50 parameters, revealed that *T. reesei* and *E. coli* coexisted stably despite the isobutanol toxicity effect. When using pretreated corn stover as substrate, various sugars in the hydrolysates were co-consumed and isobutanol production titer and yield reached 1.88 g L^−1^ and 62%, respectively. Compared to the traditional “superbug” CBP, dividing the entire isobutanol process into *T. reesei* and *E. coli* provided an optimal environment for functional expression of both the cellulase-producing pathway and sugar-conversion pathway. Moreover, the modular design could facilitate rapid adaption of the consortium system to produce a portfolio of advanced chemicals from plant biomass.

A more complicated, multi-species biofilm membrane (MBM) reactor was used to produce ethanol from undetoxified pretreated wheat straw (Brethauer and Studer, 2014) (Figure 5C). The meticulously designed MBM system maintained an optimal environment for each of the three specialists, namely *T. reesei, S. cerevisiae*, and *S. stipitis*, for cellulase production, glucose-to-ethanol conversion, and xylose-to-ethanol conversion, respectively. In MBM fermentation, a maximum of 9.8 g L^−1^ ethanol with a yield of 69% was obtained, which was substantially higher than the ethanol titer (4.6 g L^−1^) and yield (41%) obtained by co-culture of the engineered *S. cerevisiae* and *S. stipitis* using the same substrate. Despite the advantageous properties of the MBM system, limitations such as the large portion of residual cellobiose in plant biomass hydrolysates must be resolved before this system can serve as an efficient platform for economic biochemical production.

## Conclusions and Future Perspectives

Grave concerns regarding the future availability of fossil fuels, coupled with the negative environmental ramifications of the globe's current carbon footprint, is driving the initiative toward “green” fuels and chemicals. Although a step in the right direction, first-generation biorenewable products made from food crops led to concerns regarding food security. Instead, utilization of lignocellulosic biomass as the second-generation feedstock for fuel and chemical production has numerous potential environmental and societal advantages compared to its petroleum and sugar feedstock counterparts. Since 2008, global efforts from major chemical/fuel companies (e.g., POET, Raizen, etc.) have turned dozens of commercial or semi-commercial scale cellulosic ethanol plants into operation. A typical cellulosic ethanol plant has a production capacity on the order of tens of million gallons per year, which is nowhere near the total consumption rate of petroleum. As a reference, U.S. petroleum consumption was estimated to be on average, 840 million gallons per day in 2017. Despite the price fluctuation of crude oil in the past decade (in a range of $30 to $110 per barrel), bioethanol production needs to be near $70 per barrel in order to be economically competitive, which makes the co-utilization of all the sugars present in biomass hydrolysates essential. Therefore, future efforts should focus on integrating the capacities of transporting and converting all major sugars in a CCR-free manner.

Previous studies of mixed-sugar fermentation in *S. cerevisiae* were mostly isolated and had limited crossovers, which have proven problematic since intracellular metabolic flux and sugar uptake can both restrict maximal production rates. Thus, future efforts should shift toward developing robust strains for industrial biomass hydrolysate conversion *via* applying global cellular engineering strategies. Instead of focusing solely on increasing pathway flux and sugar transportation, engineering strategies should go beyond this superficial level, aiming to expand our current understanding to unveil the native regulatory mechanisms underlying non-glucose utilization in *S. cerevisiae*. For example, evolutionary engineering followed by genome re-sequencing of evolved *S. cerevisiae* strains with beneficial phenotypes elucidated that reduced glucose phosphorylation rates enabled simultaneous glucose/xylose utilization (Lane et al., 2018; Papapetridis et al., 2018). Comparative transcriptomic analysis on the natural *S. cerevisiae* YB-2625 strain isolated from bagasse and the model yeast strain S288C also revealed that multiple factors contributed to the exceptional xylose-consuming capability (Cheng et al., 2018). Some factors were rather obvious, such as down-regulation of hexokinase and genes involved in glucose-repression related transcription factors. However, other factors were quite intriguing. These included up-regulation of genes encoding antioxidant enzymes, a less than obvious link to xylose uptake and utilization. Such interventions would not have been easily discovered without a global analysis approach.

Expanding the current collection of microbial factories for the highly sought-after cosugar-utilizing phenotype presents a new frontier in the biochemical industry. A high-throughput screening method of desired characteristics must first be developed to expedite the discovery of strains possessing superior phenotypes. The increasingly prevalent microbiome and metagenomic analyses will revolutionize our ability to identify new microbial species at an unprecedented rate (Shapiro et al., 2018). Newly identified nonconventional microbes will create an urgent demand for corresponding genetic manipulation tools, specifically platform technologies that can be easily transferred from one species to another. Extension of current CRISPR-Cas technology to non-conventional yeasts will enable precise genome modification, convenient pathway engineering, and wide genetic interaction analysis (Shapiro et al., 2018). Other platform technologies, such as CRISPR-mediated base-editing platforms and RNA-seq, are beneficial for expeditious mapping of superior phenotypes to genotypes.

After extensive review of the current literatures, efficient engineering of heterogeneous consortia arises as a highly promising strategy in achieving the ultimate goal of converting lignocellulosic biomass to biofuels/biochemicals in an economically competitive manner. However, several challenges must be overcome before such systems can be adapted to commercial production. Unlike their natural counterparts, synthetic communities cannot retain long-term homeostasis, particularly under industrial harsh conditions containing inhibitors and toxins (Brenner et al., 2008). Thus, combinatorial approaches, such as evolutionary engineering combined with further development of bioreactors for long-term culturing and monitoring, should be implemented to increase robustness and fine tune the efficiency of consortia (Jia et al., 2016). Additionally, computational tools should be harnessed to develop dynamic models to facilitate discovery and understanding of variables most crucial to system performance (Purnick and Weiss, 2009; Chen et al., 2018). These models can also provide rational guidelines when designing new consortia.

In addition to efficient utilization of all sugars in biomass hydrolysates, engineering efforts should also be directed toward *in situ* fixation of the CO_2_ produced through numerous decarboxylation reactions (Cheng et al., 2018). For example, various CO_2_ conservative pathways, such as non-oxidative glycolysis (Bogorad et al., 2013), the methanol condensation pathway (Bogorad et al., 2014), reductive PPPs (rPPPs) (Guadalupe-Medina et al., 2013; Xia et al., 2017), and the 3-hydroxypropionate pathway (Mattozzi et al., 2013), have been successfully introduced into either *E. coli* or *S. cerevisiae*. To alleviate CO_2_ emissions and remove the surplus NADH generated *via* the XR/XDH pathway, synthetic rPPPs were incorporated into xylose-utilizing *S. cerevisiae* strains, resulting in higher ethanol yields and lower byproduct accumulation (e.g., glycereol and xylitol) during xylose-fermentation (Li et al., 2017; Xia et al., 2017). Implementing CO_2_ conservative pathways, in conjunction with optimization of microbial consortia in CBP *via* global engineering strategies, has the potential to expedite the development of an economic, sustainable, and environmentally-friendly lignocellulosic-based biochemical industry.

## Author Contributions

MG, DP, and ZS wrote this review manuscript together. DP wrote the CBP, and cellulase secretion sections. DP and MG wrote the introduction and conclusion sections. MG wrote the remaining parts; ZS revised and polished the entire review.

### Conflict of Interest Statement

ZS declares a financial relationship with Estose Biorenewables, LLC, a startup company that aims to provide technologies to build yeast platforms to produce nutraceuticals and pharmaceuticals from biorenewables. The remaining authors declare that the research was conducted in the absence of any commercial or financial relationships that could be construed as a potential conflict of interest.
